# Effects of Transcranial Direct Current Stimulation on Cognition, Mood, Pain, and Fatigue in Multiple Sclerosis: A Systematic Review and Meta-Analysis

**DOI:** 10.3389/fneur.2021.626113

**Published:** 2021-03-08

**Authors:** Wan-Yu Hsu, Chia-Hsiung Cheng, Theodore P. Zanto, Adam Gazzaley, Riley M. Bove

**Affiliations:** ^1^Department of Neurology, Weill Institute for Neurosciences, University of California, San Francisco, San Francisco, CA, United States; ^2^Department of Occupational Therapy and Graduate Institute of Behavioral Sciences, Chang Gung University, Taoyuan, Taiwan; ^3^Healthy Aging Research Center, Chang Gung University, Taoyuan, Taiwan; ^4^Laboratory of Brain Imaging and Neural Dynamics (BIND Lab), Chang Gung University, Taoyuan, Taiwan; ^5^Department of Psychiatry, Chang Gung Memorial Hospital, Taoyuan, Taiwan; ^6^Neuroscape, University of California, San Francisco, San Francisco, CA, United States; ^7^Department of Psychiatry, University of California, San Francisco, San Francisco, CA, United States; ^8^Department of Physiology, University of California, San Francisco, San Francisco, CA, United States

**Keywords:** cognition, mood, pain, fatigue, multiple sclerosis, transcranial direct current stimulation

## Abstract

**Background:** The study aimed to evaluate the effects of transcranial direct current stimulation (tDCS) on cognition, mood disturbance, pain, and fatigue in people with multiple sclerosis (PwMS).

**Methods:** A literature search was performed on articles published between January 1990 and May 2020 in Pubmed, Medline, and Web of Science using the following keywords and their abbreviation in combinations: multiple sclerosis and transcranial direct current stimulation. Mean effect size (ES) and 95% confidence interval were calculated for each domain of interest.

**Results:** Seventeen articles with a total of 383 PwMS were included in this analysis. For cognition, a strong effect size was found for the trial administering the Symbol Digit Modalities Test (ES: 1.15), whereas trials applying the Attention Network Test showed a negative effect size of −0.49. Moderate to strong effect sizes were observed for mood disturbance (mean ES: 0.92), pain (mean ES: 0.59), and fatigue (mean ES: 0.60). Further subgroup analyses for MS-related fatigue showed that both high and low intensities of stimulation lead to nearly the same degree of favorable effects. More pronounced effects were observed in studies administering the Fatigue Severity Scale compared with studies using other fatigue measures such as the Modified Fatigue Impact Scale.

**Conclusion:** These results provide preliminary evidence that tDCS has a favorable effect on cognitive processing speed, mood disturbance, pain, and fatigue in MS. However, the effects on cognition and fatigue vary based on the specific assessment used.

## Introduction

Multiple sclerosis (MS) is the most common non-traumatic cause of neurological disability in young adults, affecting ~1,000,000 people in the United States (1) and 2.5 million people worldwide (2). Over the disease course, a wide variety of disabling symptoms may develop, including motor and sensory disturbance, vision symptoms, cognitive impairment, mood disturbance, pain, and fatigue. These functional deficits and symptoms have a drastic impact on a patient's personal functioning, social interactions, employment, and overall quality of life. Although disease modifying therapies (DMTs) that target primarily the inflammatory immunopathology of MS can slow the development of functional disabilities (3, 4), these do not specifically alleviate symptoms such as cognitive impairment, mood disturbance, pain, and fatigue. Therefore, it is of utmost importance to develop effective and alternative approaches to symptom management.

Recently, transcranial direct current stimulation (tDCS), a form of non-invasive transcranial electrical stimulation, has been probed as a possible form of non-pharmacological intervention in several neurological and psychiatric disorders (5–7), due to its safety, portability, and potential for at-home application. tDCS modulates neuronal transmembrane potential toward hyperpolarization or depolarization by delivering weak electrical currents to the scalp, thereby altering plasticity in the stimulated brain regions (8, 9). These effects have been associated with changes in resting membrane potential, alteration of transmembrane proteins, and N-methyl-d-aspartate receptor efficiency (10, 11). Depending on whether anodal or cathodal stimulation is applied, tDCS either increases or decreases cortical excitability, respectively (12, 13), in turn affecting a wide range of behavioral measures (14, 15). Studies have reported beneficial effects of tDCS on language performance (16), learning processes (17), working memory function (18), and multitasking performance (19) in healthy adults.

Specifically in patients with MS, studies suggest that tDCS could serve as a promising tool to improve cognition (20, 21), neuropathic pain (22, 23), mood (24), and fatigue (25, 26). It has been reported that by applying daily sessions of anodal tDCS for 10 days over the dorsolateral prefrontal cortex (DLPFC) during cognitive training improved attention, information processing and executive function. Further, the improvement was sustained 6 months after last treatment (21). While studies provide intriguing evidence supporting tDCS as a therapeutic strategy for MS patients [reviewed in (27–29)], beneficial effects are not always observed. For example, in a randomized, controlled trial, 1-week tDCS application showed no measurable differences in fatigue score between stimulation and placebo interventions post stimulation (30). A study with three daily tDCS over DLPFC found no effects on mood, fatigue, or attention (22). Another study administering 10 sessions of tDCS also reported that the stimulation and control groups did not differ in standard cognitive measures after the intervention (20).

The methodological discrepancies across these trials have yielded conflicting results and therefore a lack of consensus regarding the effect of tDCS on cognitive impairment, mood disturbance, pain, and fatigue in MS. To enable more definitive conclusions regarding the potential of tDCS as a therapeutic strategy for the described MS-related domains, we performed a systematic review and meta-analysis of the available data.

## Materials and Methods

### Study Identification

Computerized searches were performed in PubMed, Medline, and Web of Science to identify pertinent studies. The search terms were “multiple sclerosis” / “MS” and “transcranial direct current stimulation” / “tDCS.” Manual searches of bibliographies of relevant reviews, book chapters, and original articles were also conducted. The searches were limited to human studies published from January 1990 to May 2020 and written in English. Articles were included when the following criteria were met: (1) original research article with a main goal to examine tDCS effects on at least one of the four domains of interest (i.e., cognition, mood, pain, fatigue); (2) the patients were adults with a diagnosis of MS; (3) reports of ≥5 participants receiving tDCS; (4) outcome measures were quantitatively reported; (5) the study included experimental and control conditions. We reviewed the full text of articles that appeared to be relevant.

### Quality Assessments

To evaluate the methodological quality of the included studies, we used a modified checklist derived from a quality screening form revised by Moher et al. (31). The quality of each study was evaluated according to the following criteria: (1) random allocation: recorded as 1 if the study pointed out that participants were randomly allocated into different groups; (2) blinding procedure: ranged from 0 to 2, where 0 represented a non-described or non-blinded procedure, and 1 and 2 indicated single-blind and double-blind procedures, respectively; (3) drop-out number: recorded as the number of participants who withdrew from the study; (4) description of baseline demographic data: recorded as 1 when provided; (5) statistical comparison between interventions: denoted as 1 if performed; (6) point estimates and measures of variability: recorded as 1 if provided; (7) adverse effects: recorded as type of the events.

### Quantitative Analyses

The relevant information from each study was extracted by one author (W.-Y. H.) using a standard data recording form that included number of participants, MS subtype, mean age, mean/median Expanded Disability Status Scale (EDSS) disease severity score, mean disease duration, stimulation protocol [i.e., duration and intensity of tDCS, targeted brain region(s), method of sham stimulation], domain(s) of measures relevant to current analysis, number of dropouts, study quality (see above), outcome measures, and post-intervention mean (M) as well as standard deviation (SD) for each outcome measure in the experimental and control groups. For studies with multiple measuring points after the intervention, the post-intervention data was based on the first measurement taken after the intervention period. A wide variety of outcome measures was found across the studies, and some evaluated multiple measures. For the purposes of this meta-analysis, the measure used to assess each study was the explicitly declared primary outcome. If the primary outcome was not clearly defined, the first outcome that was reported in the results section was chosen.

For cognition and mood, one of the studies contributed more than one trial, due to different stimulation sites (24). For fatigue, four articles contributed more than one trial because they applied the stimulation over different brain regions (24, 32, 33) or employed two studies with different design (34). For pain, SD was calculated from standard error of mean (SEM) in one study (23). For fatigue outcome measures, pooled M and SD data were calculated based on subgroup M and SEM in one study (25) and estimated from a subgroup plot in another study (26). One of the studies did not report the M and SD of their outcome measures and the data were extracted from the figures (30). The SD was calculated from SEM (32, 35) and data range (36) based on the range rule of thumb (37, 38) in three of the studies. All the extracted data were carefully checked by another author (C.-H. C.) and disagreements were resolved by discussion.

The analyses were performed with Comprehensive Meta-Analysis 3.0 software (Biostat Inc, Englewood). The standardized effect sizes and 95% confidence interval (CI) were calculated to test the results of different trials. The effect sizes were calculated based on differences between the post-treatment evaluations (22, 24, 25, 32, 33, 36, 39–42), changes relative to the baseline (23), or the mean changes between pre- and post-treatments (20, 21, 26, 30, 34, 35) in the experimental and control groups, divided by the pooled SD. Because the effect sizes from each study may be influenced by the sample sizes, a weighting factor was applied to give more weight to the studies with larger samples. Finally, the mean effect sizes were obtained after combining the weighted effect size of each study. Absolute effect sizes that ranged from 0.2 to 0.49 were considered to be small (43) and a value of 0.5 is likely to be clinically meaningful (44).

The heterogeneity across effect sizes was assessed with *Q*-statistics (45) and the *I*^2^ index (46), which is useful for assessing consistency between trials (47). When significant heterogeneity was found by *Q*-statistics or when *I*^2^ > 50%, a random effects model was applied. Otherwise, a fixed effects model was used. Begg and Mazumdar rank correlation (48) was also applied to assess the publication bias. In addition, a funnel plot (49) was used to further address publication bias. In a funnel plot, the effect size is plotted against the standard error. Studies with larger sample sizes appear toward the top of the plot, and near the mean effect size, whereas studies with smaller sample sizes appeared toward the bottom of the plot, indicating more variation in these smaller studies. In the absence of publication bias, the plot may show a symmetrical distribution. Conversely, in the presence of publication bias, the funnel plot would be asymmetrical. The Trim and Fill procedure (50), a funnel plot-derived approach aimed at identifying publication bias and adjusting the results, was applied to correct for publication bias. The significance level was set at *p* ≤ 0.05.

## Results

### Evidence Base

**Figure 1:**
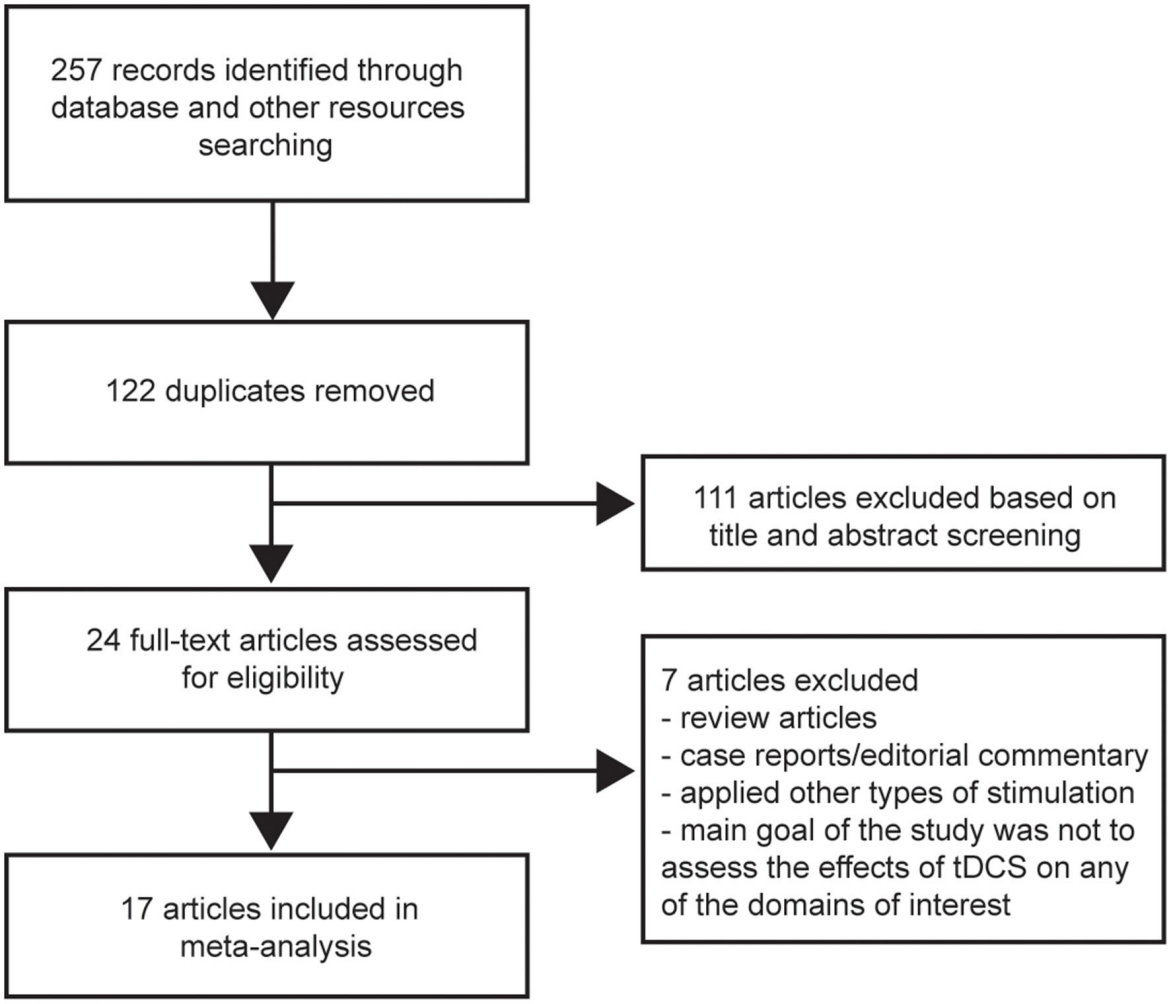
Flow diagram of study identification and inclusion as according to PRISMA guidelines.

The search yielded 257 records. After duplicates were removed, 135 articles were screened based on title and abstract. Twenty-four potentially relevant articles were obtained for full-text review; 17 articles that met our inclusion criteria were then selected (20–26, 30, 32–36, 39–42). The other seven articles were excluded for the following reasons: review articles or case reports/editorial commentary, applied other types of stimulation, or the main goal of the study was not to assess the effects of tDCS on any of the domains of interest (i.e., cognition, mood, pain, fatigue) (Figure 1). Table 1 summarizes the characteristics of the studies included in our meta-analysis. A total of 383 MS patients were involved, 251 of whom had relapsing-remitting MS. Of the 17 articles, four focused on more than one domain (22, 24, 40, 42). Four studies assessed cognition (20–22, 24). Mood and pain were measured in four (22, 24, 40, 42) and three (22, 23, 42) studies, respectively. Two studies evaluated mood status before and after the intervention, with a purpose to control for mood as a potential confounding factor (23, 30). Fourteen articles evaluated fatigue (22, 24–26, 30, 32–36, 39–42).

**Table 1:** Characteristics of each study included in the meta-analysis.

**Study**	**Number of participants (stim/sham)**	**MS subtype**	**Mean age (years) (stim/sham)**	**Mean/median EDSS (stim/sham)**	**DD (years) (stim/sham)**	**Stimulation form and protocol**	**Stimulation position and electrode size**	**Method of sham stimulation**	**Domain of measures relevant to current analysis**
Charvet et al. (20)^¶^	45 (25/20)^a^	22 RR	52.6/51.0	N/R	17.7/15.7	atDCS 1.5 mA 20 min daily for 10 days	A: L DLPFC (35 cm^2^) Ref: R DLPFC (35 cm^2^)	N/A	Cognition
Mattioli et al. (21)^¶^	20 (10/10)	20 RR	38.2/47.4	2.1/2.9	6.6/11	atDCS 2 mA 20 min daily for 10 days	A: L DLPFC (25 cm^2^) Ref: R shoulder (60 cm^2^)	30 s of stimulation at the beginning and the end of the session	Cognition
Ayache et al. (22)^*^	16 (16/16)	11 RR 4 SP 1 PP	48.9/48.9	4.25/4.25	11.8/11.8	atDCS 2 mA 20 min daily for 3 days	A: L DLPFC (25 cm^2^) Ref: R supraorbital (25 cm^2^)	Ramped down immediately after ramping up	Pain mood cognition fatigue
Mori et al. (23)	19 (10/9)	19 RR	42.8/46.3^§^	1.5/2^§^	10.1/10.3^§^	atDCS 2 mA 20 min daily for 5 days	A: primary motor cortex contralateral to the somatic painful area (35 cm^2^) Ref: contralateral supraorbital region (35 cm^2^)	Stimulator was turned off after 30 s of stimulation	Pain
Chalah et al. (24)^*^	10 (10/10)^b^	9 RR 1 SP	40.5/40.5	2.3/2.3	14/14	atDCS 2 mA 20 min daily for 5 days	(1) A: L DLPFC (25 cm^2^) Ref: R supraorbital region (25 cm^2^) (2) A: R PPC (25 cm^2^) Ref: Cz (25 cm^2^)	Ramped up for 15 s followed by 30 s of stimulation and a ramping down period of 15 s	Fatigue cognition mood
Ferrucci et al. (25)^*^	25 (25/25)	22 RR 3 SP	44.5/44.5^†^	3.2/3.2^†^	13.2/13.2^†^	atDCS 1.5 mA 15 min daily for 5 days	A: bilateral motor cortex (35 cm^2^) Ref: R deltoid (35 cm^2^)	Stimulator was turned off after 10 s of stimulation	Fatigue
Hanken et al. (26)	46 (23/23)	18 RR 28 SP	51.3/46.8^c^	4.4/3.95^c^	11.5/12.7^c^	atDCS 1.5 mA for 20 min	A: R parietal cortex (35 cm^2^) Ref: contralateral forehead (35 cm^2^)	Ramped up for 8 s followed by 30 s of stimulation and a ramping down period of 5 s, and then every 550 ms, a current of 110 μA was released	Fatigue
Saiote et al. (30)^*^	13 (13/13)	13 RR	46.8/46.8	3.5/3.5	9/9	atDCS 1 mA 20 min daily for 5 days	A: L DLPFC (35 cm^2^) Ref: contralateral forehead (90 cm^2^)	Ramped down immediately after ramping up	Fatigue
Mortezanejad et al. (32)	36 (12/12)^d^ (12/12)^e^	N/R	33.3/32.5^d^ 32.0/32.5^e^	1.75/1.37^d^ 1.46/1.37^e^	N/R	atDCS 1.5 mA 20 min daily for 6 days	(1) A: L M1 (35 cm^2^) Ref: contralateral supraorbital region (35 cm^2^) (2) A: L DLPFC (35 cm^2^) Ref: contralateral supraorbital region (35 cm^2^)	Stimulator was turned off after 30 s of stimulation	Fatigue
Tecchio et al. (33)^*^	13 (13/13)^f^ 8 (8/8)^g^	21 RR	45.8/45.8^f^ 38.1/38.1^g^	1.5/1.5^f^ 2/2^g^	7.6/7.6^f^ 13.5/13.5^g^	atDCS 1.5 mA 15 min daily for 5 days	(1) A: bilateral SI_wb_ (35 cm^2^) Ref: Oz (84 cm^2^) (2) A: bilateral SM1_hand_ 35 cm^2^ Ref: under the chin (84 cm^2^)	4 s of stimulation at the beginning and the end of the session	Fatigue
Charvet et al. (34)	35 (15/20)^h^ 27 (15/12)^i^	18 RR^h^ 13 RR^i^	53.4/51.0^h^ 44.8/43.4^i^	6/4^h^ 6/3.5^i^	15.6/15.7^h^ 15.8/13.3^i^	^h^atDCS 1.5 mA 20 min daily for 10 days ^i^atDCS 2 mA 20 min daily for 20 days	A: L DLPFC (25 cm^2^)	^i^Ramp up to 2.0 mA and back down during the first and last minutes of the session	Fatigue
Fiene et al. (35)^*^	15 (15/15)	14 RR 1 SP	43.2/43.2	3.54/3.54	9.63/9.63	atDCS 1.5 mA for a mean duration of 27.29 min	A: L DLPFC (25 cm^2^) Ref: R shoulder (35 cm^2^)	Current turned off after 30 s with a ramp-down of 15 s	Fatigue
Porcaro et al. (36)^*^	18 (18/18)	18 RR	44.5/44.5	1.1/1.1	6.9/6.9	atDCS 1.5 mA 15 min daily for 5 days	A: bilateral SI_wb_ (35 cm^2^) Ref: Oz (70 cm^2^)	4 s of stimulation at the beginning and the end of the session	Fatigue
Cancelli et al. (39)^*^	10 (10/10)	10 RR	43.2/43.2	0.9/0.9	6.6/6.6	atDCS 1.5 mA 15 min daily for 5 days	A: bilateral SI (35 cm^2^) Ref: Oz (70 cm^2^)	4 s of stimulation at the beginning and the end of the session	Fatigue
Chalah et al. (40)^*^	11 (11/11)	10 RR 1 SP	43.9/43.9	3.14/3.14	6.3/6.3	atDCS 2 mA 20 min daily for 5 days	A: L DLPFC (35 cm^2^) Ref: R DLPFC (35 cm^2^)	Ramped up for 15 s followed by 30 s of stimulation and a ramping down period of 15 s	Fatigue mood
Tecchio et al. (41)^*^	10 (10/10)	7 RR 1 SP 2 PP	45.8/45.8	1.5/1.5	7.1/7.1	atDCS 1.5 mA 15 min daily for 5 days	A: bilateral SI (35 cm^2^) Ref: Oz (70 cm^2^)	4 s of stimulation at the beginning and the end of the session	Fatigue
Workman et al. (42)^*^	6 (6/6)	6 RR	46.7/46.7	N/R	N/R	atDCS 2 mA 20 min daily for 5 days	A: M1 representation of the more-affected leg (35 cm^2^) Ref: contralateral supraorbital region (35 cm^2^)	Ramped up to 2 mA and then the current was set to 0 mA	Pain fatigue mood

*stim, stimulation group; sham, sham group; EDSS, Expanded Disability Status Scale; DD, disease duration; RR, relapsing-remitting; SP, secondary-progressive; PP, primary-progressive; atDCS, anodal transcranial direct current stimulation; A, anode; Ref; reference; SI, primary somatosensory cortex; SI_wb_, whole body somatosensory areas; SM1_hand_, hand sensorimotor areas; PPC, posterior parietal cortex; M1, primary motor cortex; DLPFC, dorsolateral prefrontal cortex; R, right; L, left; N/R, not reported; N/A not applicable*.

¶ *tDCS was paired with cognitive training*.

* *Cross-over design*.

§ *Data calculated from Mori et al. (23), Table 1*.

† *Data calculated based on 23 participants included in the final analysis in Ferrucci et al. (25), Table 1*.

a *Participants in the control group did not receive either tDCS or sham stimulation*.

b *Data from 10 participants included in the final analysis in Chalah et al. (24)*.

c *Data calculated based on 40 participants included in the final analysis in Hanken et al. (26), Table 4*.

d *Participants in M1 group*.

e *Participants in L DLPFC group*.

f *Participants in SI_wb_ group*.

g *Participants in SM1_hand_ group*.

h *Study 1, open-label study. Twenty participants only participated in cognitive training and did not receive either tDCS or sham stimulation*.

i *Study 2, randomized controlled trial*.

### Intervention

These studies employed different study designs. Two studies were designed as single session trials (26, 35). Ten studies applied the stimulation at an intensity lower than 2 mA (20, 25, 26, 30, 32, 33, 35, 36, 39, 41). Target stimulation regions included motor cortex (23, 25, 32, 42), dorsolateral prefrontal cortex (20–22, 24, 30, 32, 34, 35, 40), primary somatosensory cortex (33, 36, 39, 41), sensorimotor cortex (33) and parietal cortex (24, 26).

### Outcome Measures

A variety of outcome measures was used in the selected articles. For cognition, Attention Network Test (22, 24), Symbol Digit Modalities Test (21) and Brief International Cognitive Assessment for MS (20) were performed. For mood, Hospital Anxiety and Depression Scale (22, 24, 40) and Beck Depression Inventory (42) were included. Pain was assessed with Visual Analog Scale (22, 23, 42). Fatigue was assessed using the Modified Fatigue Impact Scale in eight trials (30, 33, 36, 39–41, 51); other outcome measures for fatigue included Fatigue Impact Scale (25), vigilance task (26), Fatigue Severity Scale (24, 32), Patient-Reported Outcomes Measurement Information System-fatigue short form (34), simple reaction time task (35), and fatigue index (42).

### Methodological Quality

Table 2 shows the quality assessment results of the included studies. Random allocation was achieved in all the studies except two trials (20, 34). Most of the studies were of double-blind (21–26, 30, 32–34, 36, 39–42) or single-blind (35) design. Baseline demographic data were described in all the studies. Six studies had drop-outs (20, 24, 25, 33, 34, 41). Statistical comparisons were completed in all the articles; however, one study did not provide point estimates and measures of variability (30). Eight studies reported adverse events. These included skin reaction, insomnia, tingling, itching, phosphene, burning sensation, head pain or pressure, difficulty concentrating, facial muscle twitching, nausea, fatigue, and iron taste (21, 22, 24, 25, 30, 32, 34, 40). One study (23) reported no adverse events.

**Table 2:** Quality assessment for studies included in the meta-analysis.

**Study**	**Random allocation**	**Blinding**	**Baseline demographic data**	**Drop-outs**	**Between conditions statistical comparison**	**Point estimates and variability**	**Adverse effects**
Charvet et al. (20)	0	0	1	1	1	1	N/R
Mattioli et al. (21)	1	2	1	0	1	1	Itchiness, pain, burning, warmth, pinching, fatigue, iron taste
Ayache et al. (22)	1	2	1	0	1	1	Insomnia, nausea, headache, phosphene
Mori et al. (23)	1	2	1	0	1	1	None
Chalah et al. (24)	1	2	1	2	1	1	Insomnia, headache
Ferrucci et al. (25)	1	2	1	2	1	1	Skin reaction
Hanken et al. (26)	1	2	1	0	1	1	N/R
Saiote et al. (30)	1	2	1	0	1	0	Headache, skin sensation
Mortezanejad et al. (32)	1	2	1	0	1	1	Tingling, itching
Tecchio et al. (33)	1	2	1	2	1	1	N/R
Charvet et al. (34)	0	0	1	0	1	1	Tingling, itching, burning sensation, head pain or pressure, difficulty concentrating, facial muscle twitching, nausea
	1	2	1	2	1	1	
Fiene et al. (35)	1	1	1	0	1	1	N/R
Porcaro et al. (36)	1	2	1	0	1	1	N/R
Cancelli et al. (39)	1	2	1	0	1	1	N/R
Chalah et al. (40)	1	2	1	0	1	1	Phosphene, sleep disturbance
Tecchio et al. (41)	1	2	1	1	1	1	N/R
Workman et al. (42)	1	2	1	0	1	1	N/R

*N/R, not reported*.

### Meta-Analysis

Table 3 summarizes the domains of measures, outcome measures, the number of participants in the post-treatment evaluations, mean and SD, and effect size of each study.

**Table 3:** Summary of the effect sizes.

**Domain of measures**	**Study**	**Outcome measures**	**Nexp/Nctrl**	**Mexp/Mctrl**	**SDexp/SDctrl**	**ES**
Cognition	Charvet et al. (20)	BICAMS	24/20	0.09/0.09	0.47/0.47	0.00
	Mattioli et al. (21)	SDMT	10/10	8.8/−0.1	8.6/6.7	1.15
	Ayache et al. (22)	ANT (alertness)	16/16	52.1/58.8	36/66	−0.12
	Chalah et al. (24)	ANT^a^ (mean reaction time)	10/10	660.2/620.6	29.7/34	−1.24
		ANT^b^ (mean reaction time)	10/10	634.7/620.6	26.2/34	−0.46
Mood	Ayache et al. (22)	HADS_total_	16/16	13.6/14.5	5.8/6.5	0.14
	Chalah et al. (24)	HADS_anxiety_a	10/10	2.8/3.8	0.5/1.0	1.26
		HADS_anxiety_b	10/10	2.0/3.8	0.5/1.0	2.27
	Workman et al. (42)	BDI	6/6	11.5/9.8	12.1/7.0	0.17
Pain	Ayache et al. (22)	VAS	16/16	43.1/50.3	26.2/19.7	0.31
	Mori et al. (23)	VAS	10/9	45.5/89.3	34.7/25.8^c^	1.42
	Workman et al. (42)	VAS	6/6	11.3/18.8	12.8/34.5	0.28
Fatigue	Ayache et al. (22)	MFIS	16/16	49.0/47.4	15.2/17.7	−0.09
	Chalah et al. (24)	FSS^a^	10/10	3.3/3.9	0.4/0.5	1.32
		FSS^b^	10/10	3.8/3.9	0.5/0.5	0.20
	Ferrucci et al. (25)	FIS	23/23	46.3/46.3^d^	21.6/26.9^d^	0.00
	Hanken et al. (26)	Vigilance task	20/20	−20/35^e^	84.71/71.46^e^	0.70
	Saiote et al. (30)	MFIS	13/13	0.5/−3^f^	5.4/4.5^f^	−0.7
	Mortezanejad et al. (32)	FSS^g^	12/12	3.79/4.71	0.51/0.51^c^	1.80
		FSS^h^	12/12	3.55/4.71	1.07/0.51^c^	1.38
	Tecchio et al. (33)	MFIS^i^	13/13	31.0/34.7	12.0/10.4	0.33
		MFIS^j^	8/8	42.1/52.1	17.2/22.0	0.50
	Charvet et al. (34)	PROMIS-Fatigue short form^k^	15/20	−2.5/−0.2	7.4/5.3	0.36
		PROMIS-Fatigue short form^l^	15/12	−5.6/0.9	8.9/1.9	0.95
	Fiene et al. (35)	Simple reaction time task	15/15	−2.76/6.99	14.0/18.5^c^	0.59
	Porcaro et al. (36)	MFIS	18/18	32.5/41.4	11.5/10.7^m^	0.80
	Cancelli et al. (39)	MFIS	10/10	27.6/46.0	19.4/18.6	0.96
	Chalah et al. (40)	MFIS	11/11	39.27/41.73	22.0/19.3	0.11
	Tecchio et al. (41)	MFIS	10/9	31.0/34.8	4.0/3.5	1.00
	Workman et al. (42)	Fatigue index	6/6	50.1/72.3	11.9/11.3	1.91

*N, number of patients in the post-treatment evaluation; exp, experimental group; ctrl, control group; M, mean; SD, standard deviation; ES, effect size; ANT, Attention Network Test; SDMT, Symbol Digit Modalities Test; BICAMS, Brief International Cognitive Assessment for MS; HADS, Hospital Anxiety and Depression Scale; BDI, Beck Depression Inventory; VAS, Visual Analog Scale; FIS, Fatigue Impact Scale; PROMIS, Patient-Reported Outcomes Measurement Information System; MFIS, Modified Fatigue Impact Scale; FSS, Fatigue Severity Scale*.

a *Left dorsolateral prefrontal group*.

b *Right posterior parietal cortex group*.

c *Data calculated from standard error of the mean*.

d *Pooled data were calculated based on subgroup mean and standard error of mean listed in Table 2, Ferrucci et al. (25)*.

e *Data from Figure 5, Hanken et al. (26)*.

f *Data from Figure 3, Saiote et al. (30)*.

g *Motor cortex group*.

h *Dorsolateral prefrontal cortex group*.

i *Data for SI_wb_ group*.

j *Data for SM1_hand_ group*.

k *Data from open-label study*.

l *Data from randomized controlled trial*.

m *Data calculated from data range based on range rule of thumb*.

#### Cognition

**Figure 2:**
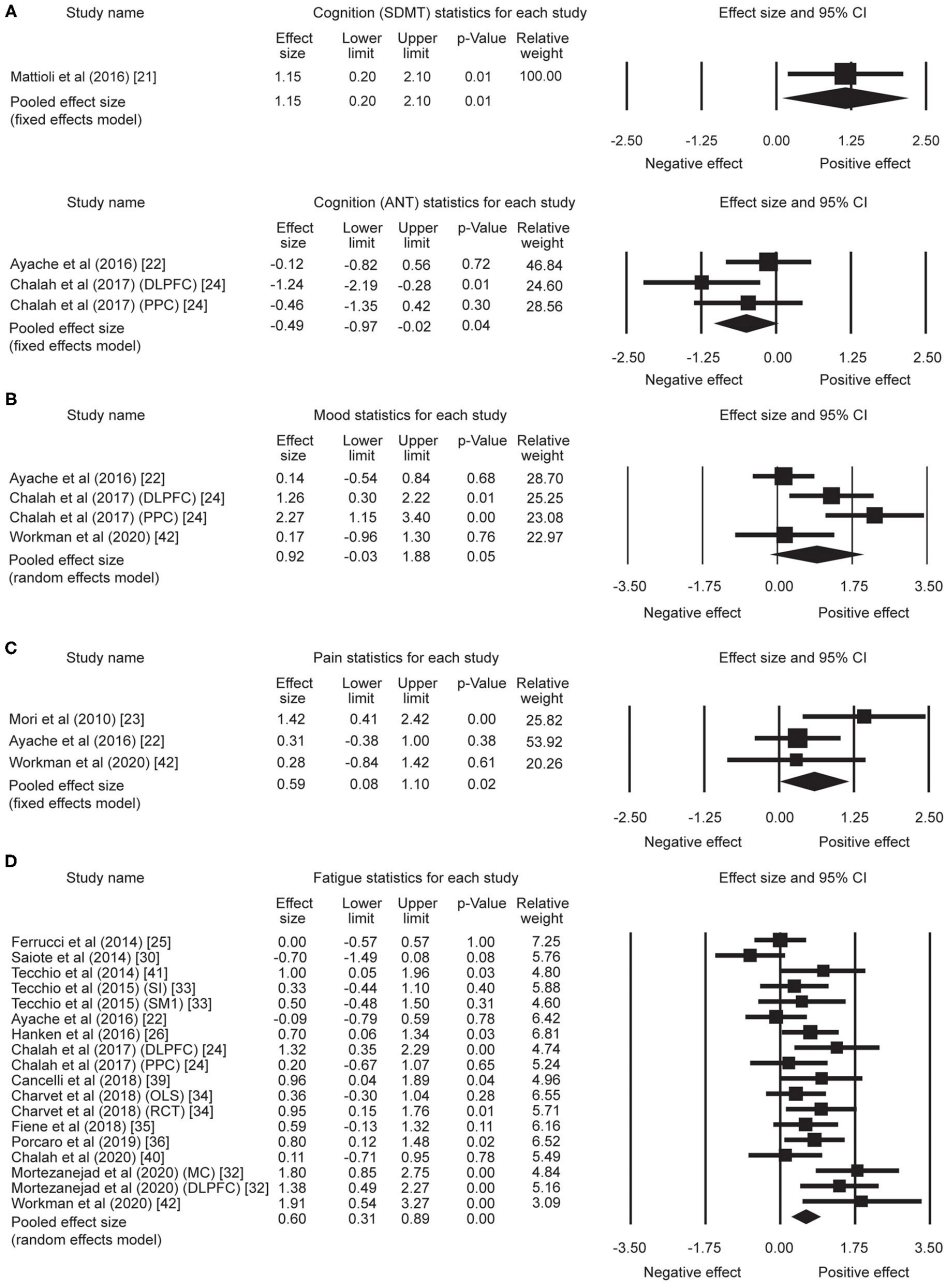
Statistical summary and forest plot of effect sizes for **(A)** cognition, **(B)** mood, **(C)** pain, and **(D)** fatigue outcome measures. SDMT, Symbol Digit Modalities Test; ANT, Attention Network Test; CI, confidence interval; DLPFC, dorsolateral prefrontal cortex; PPC, posterior parietal cortex; SI, whole body somatosensory areas; SM1, hand sensorimotor areas; OLS, open-label study; RCT, randomized controlled trial; MC, motor cortex.

**Figure 3:**
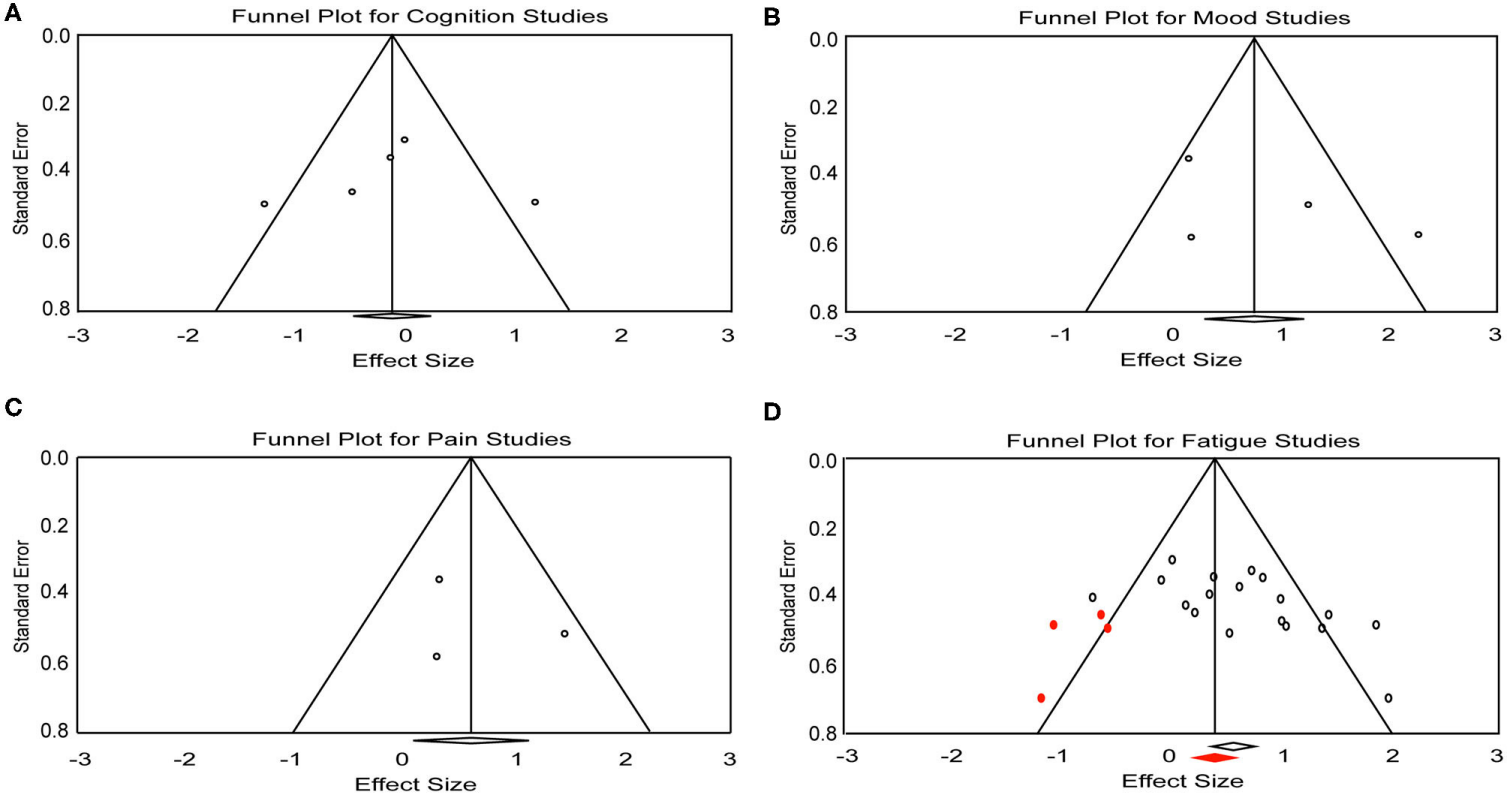
Funnel plot for **(A)** cognition, **(B)** mood, **(C)** pain, and **(D)** fatigue studies included in the meta-analysis. Red dots represent the imputed missing studies. Red rhombus shows the adjusted mean effect size.

A total of five effect sizes was obtained from four articles with 90 patients (Table 3). Since it has been demonstrated that tDCS effects on cognition are task- and cognitive domain-specific (52, 53), we divided the studies into two separate analyses based on the cognitive tasks evaluated: [Symbol Digit Modalities Test (SDMT) vs. Attention Network Test (ANT)], given that SDMT is the most widely used measure of information processing speed in MS (54, 55) and ANT is the most commonly administered task in the five trials. One study that administered the SDMT as part of the Brief International Cognitive Assessment for MS but only reported composite scores (20) was excluded from the subsequent analyses. Therefore, only four trials with a total of 46 patients were included in task-specific analyses. The analyses revealed an effect size of 1.15 (95% CI, 0.20–2.10, *p* = 0.01) for the trial administering the SDMT (21). Mean effect size for trials that applied ANT was −0.49 (95% CI, −0.97 to −0.02, *p* = 0.04) (Figure 2A). We did not find heterogeneity among the studies that applied ANT (*Q* = 3.42, *I*^2^ = 41.55, *p* = 0.18). Heterogeneity analysis was not applicable for SDMT since only one trial was included. Publication bias was not found based on rank correlation (tau = −0.30, *p* = 0.46) when considering all five trials investigating tDCS effects on cognition. The funnel plot resembles an inverted symmetrical funnel, which confirmed that publication bias is absent (Figure 3A).

#### Mood

Four effect sizes were obtained from three articles with a total of 32 patients for mood. A strong mean effect size of 0.92 (95% CI, −0.03–1.88, *p* = 0.05) (Figure 2B) was found. There was heterogeneity across the studies (*Q* = 12.08, *I*^2^ = 75.17, *p* = 0.007). The results of rank correlation (tau = 0.33, *p* = 0.49) and the symmetrical funnel plot (Figure 3B) indicate that publication bias did not seem to affect the validity of the overall effect size obtained by the meta-analysis of mood. Two studies evaluating mood as a control, rather than outcome variable, were not included in the meta-analysis (23, 30). Mood status was measured by Chalah et al. (40) but the effect sizes could not be determined since point estimates for the control group were not reported.

#### Pain

Three effect sizes were determined for pain from three articles with a total of 41 patients. A moderate mean effect size of 0.59 (95% CI, 0.08–1.10, *p* = 0.02) (Figure 2C) was discovered. We did not find heterogeneity among the studies (*Q* = 3.49, *I*^2^ = 42.82, *p* = 0.17). Publication bias was not found by either rank correlation (tau = 0.00, *p* = 1.00) or the funnel plot (Figure 3C).

#### Fatigue

A total of 18 effect sizes were extracted from 14 articles (with 291 patients), and the mean effect size was 0.60 (95% CI, 0.31–0.89, *p* < 0.001) (Figure 2D). Heterogeneity was observed across studies (*Q* = 38.45, *I*^2^ = 55.79, *p* = 0.002). Publication bias was discovered by rank correlation (tau = 0.39, *p* = 0.02) and an asymmetrical funnel plot showing a higher concentration of studies on one side of the mean than the other (Figure 3D). Therefore, a planned Trim and Fill procedure (50) was applied to impute missing studies. After adjusting for missing studies, a mean effect size of 0.39 was found.

Since a larger number of effect sizes (i.e., 18) was extracted for fatigue, we explored whether other variables would influence the measured effect. To achieve this, we performed subgroup analyses based on stimulation intensity (low: <2 mA vs. high: ≥2 mA) and outcome measures [Fatigue Severity Scale (FSS) vs. Modified Fatigue Impact Scale (MFIS) vs. other outcomes for fatigue] that were applied in the studies. The subgroup analysis of stimulation intensity revealed a mean effect size of 0.62 (95% CI, 0.05–1.19, *p* = 0.03) for six trials from five studies (22, 24, 34, 40, 42) with a “high” intensity (i.e., ≥2 mA). Mean effect size for 12 trials from 10 studies (25, 26, 30, 32–36, 39, 41) with “low” intensity (i.e., <2 mA) was 0.60 (95% CI, 0.25–0.95, *p* = 0.001). For the analysis of outcome measures, a mean effect size of 1.14 (95% CI, 0.68–1.60, *p* < 0.001) was found for FSS [four trials (24, 32)]. The mean effect sizes for MFIS [eight trials (22, 30, 33, 36, 39–41)] and other fatigue outcomes [six trials, including Fatigue Impact Scale (25), vigilance task (26), Patient-Reported Outcomes Measurement Information System-fatigue short form (34), simple reaction time task (35), and fatigue index (42)] were 0.31 (95% CI, 0.03–0.60, *p* = 0.03) and 0.53 (95% CI, 0.23–0.82, *p* < 0.001), respectively.

## Discussion

The results of this meta-analysis suggest that tDCS might be helpful in improving cognition (processing speed), mood disturbance, pain, and fatigue in MS. There has been increasing interest in treatment strategies to improve cognitive impairment (56). Here, we found a strong effect size of 1.15 for the trial that administered SDMT, and a negative effect for the trials that used ANT (effect size = −0.49). The results suggest that tDCS-induced cognitive improvement is task-specific or cognitive domain-specific. However, the findings should be interpreted with caution given the small sample size. SDMT is a widely used test in MS clinical trials and mainly evaluates information processing speed and immediate visual memory recall. Since cognitive processing speed is the most commonly affected cognitive domain (57, 58), it is possible that the test is more sensitive to detect cognitive improvements, including changes induced by tDCS. It is unclear why the performance of ANT was not improved by tDCS. One possibility is that the stimulation duration might not have been optimal. For instance, in the trial that administered SDMT and showed positive effects, 10 sessions of stimulation were applied (21). However, in studies using ANT as an outcome, no more than five sessions of stimulation were employed (22, 24). Study design may also affect the results: the study administering SDMT delivered tDCS during cognitive training, whereas the studies using ANT did not pair the stimulation with cognitive tasks. Another possible explanation is that baseline cognitive performance is a critical factor in determining whether tDCS—or any cognitive intervention—enhances cognitive performance (59, 60). Since most of the studies included in this meta-analysis did not specifically recruit patients with cognitive impairment, the heterogeneity in cognitive performance across participants may have affected the results. Further investigation with more homogeneous patient populations, different stimulation protocols, and cognitive assessments is needed to draw a conclusion regarding the optimal stimulation protocol and the effect of tDCS on different dimensions of cognition.

A strong mean effect size of 0.92 was discovered for mood disturbance. Further, studies that measured pain showed a mean effect size of 0.59, which is clinically meaningful (44). Neuropathic pain is one of the most common symptoms (61) and it is thought to be a consequence of maladaptive plastic changes within the nociceptive system which alters nociceptive signal processing (62). Studies have suggested that pain decreased by tDCS may be the result of functional changes in brain structures that are critical in pathogenesis of neuropathic pain (22, 23). By acting on pain-related corticosubcortical and corticocortical pathways, tDCS modulates perception of pain and reduces chronic neuropathic pain. However, further studies are warranted to better differentiate tDCS effects on neuropathic and nociceptive pain. While the results suggested beneficial effects of tDCS on mood disturbance and pain, the findings should be viewed conservatively since the sample size is small (mood: 32 patients; pain: 41 patients).

The mean effect size for fatigue was 0.60. A subgroup analysis was conducted to explore whether stimulation intensity and outcome measures being applied would influence the measured effect for fatigue. Both high and low intensities of stimulation demonstrated moderate effect sizes (high: effect size = 0.62; low: effect size = 0.60), suggesting that high and low intensities could yield nearly the same level of favorable effects on fatigue. Interestingly, graded stimulation effects were reported previously, where a larger learning effect was observed in healthy adults when the stimulation is applied at a higher intensity (63). Given that chronic inflammatory activity (64) and central inflammation (65) are related to synaptic plasticity, it is possible that how the brain responds to the tDCS intervention is altered. In this scenario, stimulation could lead to qualitatively different outcomes in intact vs. dysfunctional neural circuits. In contrast to the findings in healthy adults, we found that both high and low stimulation intensities relieved fatigue, with a similar degree of effect. Subgroup analysis of outcome measures demonstrated a relatively higher effect size for trials using the FSS (effect size = 1.14) than those using the MFIS (effect size = 0.31) and other outcomes assessments (effect size = 0.53), indicating that the FSS may be more sensitive to detect changes in fatigue induced by tDCS. Both the FSS and MFIS are widely used in assessing fatigue, but the item contents of the two scales are different. While the FSS primarily targets physical aspects of fatigue, MFIS measures physical, cognitive and psychosocial fatigue. Since the two scales measure different aspects of fatigue (66), the observed larger effect size for trials using the FSS suggests that tDCS effects may be more beneficial to treat physical fatigue. Physical fatigue in MS is associated with a progressive disease course and greater physical disability (67). Often, the impact of physical dysfunction on daily activities can be recognized more easily than that of mental fatigue. However, it is unclear how reliably a patient can actually distinguish between physical and mental fatigue, since perceived mental or physical fatigue does not correlate with objective measures of cognitive or physical performance (68, 69). Thus, further studies in a larger population are required to better determine the most sensitive outcome measures for detecting tDCS effects on fatigue.

One important consideration for this systematic review and meta-analysis is the methodological quality of the selected studies. Most of the trials included did achieve random allocation, and reported control groups and blinding procedures. However, two studies measuring tDCS effects on fatigue provided no point estimates or measures of variability, and these data were estimated from their figures (26, 30). The influence of non-precise data on the mean effect size cannot be fully excluded. Further, possible publication bias was detected in studies for fatigue. Although a Trim and Fill procedure (50) was performed to adjust the mean effect size, the results obtained in the present meta-analysis must be viewed conservatively. Despite the funnel plot and rank correlation analyses both indicating there was no publication bias in the studies for cognition, mood and pain, bias could not be fully excluded since the small number of trials included could limit the bias detection.

While tDCS is generally thought to be safe for both healthy adults and clinical populations, and no severe adverse effects have been reported, investigators should adhere to safety guidelines (70) and conduct follow-up assessments to monitor longer-term risks and benefits. In addition to safety concerns, several crucial questions should be addressed in future studies with proper experimental design. First, it is essential to elucidate the underlying neural mechanisms of positive effects on cognition, mood, pain, and fatigue induced by the tDCS. Second, further investigation is needed for optimizing stimulation protocols and finding the most effective parameters to apply tDCS as a treatment approach for MS. Third, studies with subgroups that are varied in subtypes of MS and clinical severity are necessary to identify the subgroups of patients most likely to benefit from tDCS. Studies have demonstrated that the efficacy of non-invasive electrical stimulation is correlated with the magnitude of the electric field that reaches the targeted brain area, which highlights the importance of anatomical variability and individualizing stimulation protocols (71–73). Thus, inter-individual variability in response to tDCS should be taken into account.

Some limitations exist in the review. First, it is difficult to estimate potential confounders such as regimens and types of DMTs, disease evolution profiles and effects of medicinal products. In the studies included in the meta-analysis, mood, pain, and fatigue were mainly measured with patient-reported outcome measures, which have very little or no motor component involved. For cognition, a motor component was involved in performing the task. However, how motor function, and other factors such as spasticity and fatigue, could have influenced the cognitive performance was not explicitly discussed. Second, we may have missed relevant studies that were published in non-English languages. Third, the findings of the current study should be taken with caution given the relatively small sample size and the repeated analyses in the same domain (e.g., ANT task) with the same patient population. The fact that relapsing-remitting MS was the majority population also makes it difficult to provide information about differences in treatment response between MS subtypes. Finally, methodological variations existed between the selected studies with respect to outcome measures, patient inclusion criteria, experimental design (e.g., cross-over vs. parallel design), and tDCS protocols. For instance, in studies measuring fatigue, the number of stimulation sessions varied across trials, with a range from single session to 20 sessions. Previous studies have reported that repeated sessions of tDCS can result in cumulative effects (74, 75). Although trials applied 20 sessions of tDCS (34) did not show a larger ES (0.95) compared to trials with five or six sessions of stimulation (ES ranging from −0.7 to 1.91), the influence of heterogeneity across the studies on the effect estimation cannot be ruled out. Stimulation timing (“online” vs. “offline”) and intervals between stimulation sessions are also critical factors that may affect the observed effects. However, subgroup analyses based on these factors are not suitable given the low number of total studies included, which limited us to simply determine the different degrees of the effect generated by timing of the stimulation and stimulation intervals.

In conclusion, this meta-analysis suggests preliminary evidence of favorable effects of tDCS on cognition, mood disturbance, pain, and fatigue in MS. For cognition, tasks targeting cognitive aspects including processing speed, may be more suitable to reflect tDCS-enhanced cognitive performance. For fatigue, applying high and low intensities of stimulation generate nearly the same grade of beneficial effects, and a relatively higher effect size was noted in studies using FSS as an outcome, suggesting that it may be more sensitive in capturing tDCS-induced changes in fatigue. Further well-designed studies are necessary to determine the neural plasticity changes induced by tDCS, optimize stimulation protocol and identify the subgroups of patients who would benefit most.

## Data Availability Statement

The raw data supporting the conclusions of this article will be made available by the authors, without undue reservation.

## Author Contributions

W-YH: conceptualization, methodology, data analysis, visualization, and manuscript writing. C-HC: data curation and content curation. TZ, AG, and RB: conceptualization and manuscript editing. All authors: contributed to the article and approved the submitted version.

## Conflict of Interest

RB has received research support from the National Multiple Sclerosis Society, the Hilton Foundation, the California Initiative to Advance Precision Medicine, the Sherak Foundation and Akili Interactive. RB has also received personal compensation for consulting from Alexion, Biogen, EMD Serono, Novartis, Pear Therapeutics, Roche Genentech and Sanofi Genzyme. AG is a scientific advisor for Neuroelectrics and Halo Neuroscience, companies that produces tES devices, and co-founder, shareholder, BOD member, and advisor for Akili Interactive Lab, a company that produces therapeutic video games. TZ is a scientific advisor for HUMM, a company that produces tES devices. The remaining authors declare that the research was conducted in the absence of any commercial or financial relationships that could be construed as a potential conflict of interest.
